# Does medical students’ personality have an impact on their intention to show empathic behavior?

**DOI:** 10.1007/s00737-018-0837-y

**Published:** 2018-04-06

**Authors:** Tamara Seitz, Angelika S. Längle, Charles Seidman, Henriette Löffler-Stastka

**Affiliations:** 1 Department of Infectious Diseases and Tropical Medicine, SMZ Süd Wien, Vienna, Austria; 2 Department of Psychoanalysis und Psychotherapy, and Teaching Center/Postgraduate Unit, Medical University of Vienna, Währingerstraße 18-20, 1090 Vienna, Austria; 3 Columbia University, New York, NY 10027 USA

**Keywords:** Empathy, NEO-FFI, Personality, Medical students, Doctor-patient communication

## Abstract

Several studies have demonstrated a correlation between specific personal traits and empathy. However, it is not clear if persons with certain personality traits lack the intent to show empathic behavior or if other factors independent of their intent are affecting their empathic behavior. To answer this question, we asked 132 medical students to fill out questionnaires evaluating the General Intention to Show Empathic Behavior (GISEB) and the five personality traits measured by NEO Five-Factor Inventory (NEO-FFI). Additionally, we evaluated the influence of other factors, such as age, gender, curricular progress (second versus fourth year), and preferred specialization after graduation. We performed a Pearson’s correlation and a regression analysis. Results indicate that the five personality traits and gender have little influence on the General Intention (GISEB), only extraversion (*r* = .221, 95% CI [.013–.394], *p* = .027), and agreeableness (*r* = .229, 95% CI [.021–.428], *p* = .022) correlated with the intention. The only predictor for General Intention (GISEB) was curricular progress (*β* = − .27, *p* < .05), showing a decrease of General Intention to Show Empathic Behavior from second to fourth year of university (*U* = 1203.5, *p* = .002). A further finding indicates that gender and personality influence the students’ wish of specialization after graduation: Agreeableness (*F*(12, 53) = 2.376, *p* = .016) impacted the preferred specialization. Our study demonstrated that medical students’ personality might not notably impact the intention to show empathic behavior. Further research is needed to investigate moderating effects.

## Introduction

Empathy is a crucial skill for every physician. Recent literature has shown that physician’s level of empathy correlates with patient satisfaction (Winefield and Chur-Hansen 2000; Schmid Mast et al. 2004; Hojat et al. 2011; Derksen et al. 2013), patient compliance (Winefield and Chur-Hansen 2000; Schmid Mast et al. 2004; Hojat et al. 2011), and clinical outcome (Winefield and Chur-Hansen 2000; Derksen et al. 2013). It was additionally demonstrated that empathy is both learnable and trainable (Drdla and Löffler-Stastka 2016), meaning its teaching is an essential duty of every medical university. However, it was often found that the level of empathy was not only the result of the quality of the training, but heavily contingent on the student’s personality as well. An effective way to measure the personality structure is the Big Five personality model (NEO Five-Factor Inventory-3 (NEO-FFI)) (McCrae and Costa 2012), which include the five personality traits of Openness, Conscientiousness, Extraversion, Agreeableness, and Neuroticism. We found seven studies investigating the relationship between the NEO-FFI and empathy among Japanese, German, Spanish, Portuguese, and American university students. The level of empathy was evaluated by different questionnaires: Del Barrio et al. (2004), Nettle (2007), and Wakabayashi and Kawashima (2015), and Melchers et al. (2016) measured the level of empathy by the Empathy Quotient (Baron-Cohen and Wheelwright 2004). All four studies showed a significant association between Agreeableness (Del Barrio et al. 2004; Nettle 2007; Wakabayashi and Kawashima 2015; Melchers et al. 2016), two between Extraversion (Wakabayashi and Kawashima 2015; Nettle, 2007), two between Conscientiousness (Del Barrio et al. 2004; Melchers et al. 2016), and one between Openness (Del Barrio et al. 2004) and Empathy Quotient score. Other authors (Magalhães et al. 2012; Costa et al. 2014) measured the level of empathy using the Jefferson Scale of Physician Empathy (JSPE) (Kane et al. 2007), and both showed a significant correlation between Agreeableness and Openness and the Jefferson Scale of Physician Empathy score. Others (Lourinho and Severo 2013; Melchers et al. 2016) used the Interpersonal Reactivity Index (Davis 1983), demonstrating a correlation between Agreeableness (Lourinho and Severo 2013; Melchers et al. 2016), Conscientiousness (Melchers et al. 2016) and Openness (Lourinho and Severo 2013) and Interpersonal Reactivity Index score. Inconsistent findings may reflect the use of different questionnaires; regardless they emphasize the need for further conceptual reflection. In the subject-specific literature, much is being investigated and discussed on the subject of “empathy” (Pedersen 2009, p. 307). The researchers in this area are confronted with a number of problems, as various authors describe (see Pedersen 2009; Stepien and Baernstein 2006). A single, uniform definition of empathy is still a contested point (see Stepien and Baernstein 2006; Preusche 2013; Preusche and Wagner-Menghin 2013). Finally, the numerous survey methods do not clearly refer to a concise definition of empathy. Moreover, the interventions to increase empathy often seem to have no solid theoretical foundation (see Stepien and Baernstein 2006). The distinction between attitudes towards empathic behavior and empathic behavior per se is often neglected (see Preusche 2013).

Empathy consists of different aspects and processes (e.g., Batson 2011; Decety and Jackson 2004; Ickes 1993; Levenson and Ruef 1992; Zaki et al. 2008): cognitive, emotional, and unconscious influences (Knaus et al. 2016), self-other distinction, empathic behavior, and also some form of willingness—we are not constantly and unwillingly empathizing with everyone we see, a point stressed by de Vignemont (2006). Although it seems quite common to speak of empathy as an automatic reaction, it has to be assumed that it cannot be so (de Vignemont 2006; Lamm and Silani 2014).

### One definition of empathy that these authors prefer is as follows:

“The state of empathy, or being empathic, is to perceive the internal frame of reference of another with accuracy, and with the emotional components and meanings which pertain thereto, as if one were the person, but without ever losing the “as if” condition. Thus it means to sense the hurt or the pleasure of another as he senses it, and to perceive the causes thereof as he perceives them, but without ever losing the recognition that it is as if I were hurt or pleased, etc. If this “as if” quality is lost, then the state is one of identification” (Rogers 1959, pp. 210–211). Rogers’ (1959, p. 210) quote “Thus it means to sense the hurt or the pleasure of another as he senses it […]” implies that a physician should not always be empathic. It depends strongly on the situation. While a physician should be empathic while having a conversation with a patient, the same empathy is unnecessary while performing surgery. In contrast, a psychotherapist should be highly empathic all along a session (compare Mercer and Reynolds 2002).

However, other authors focus on aspects such as environmental factors and personality traits and see empathy as a kind of process (Preusche 2013) or action. According to Ajzen (1991), personality traits and demographic variables represent background factors which indirectly influence the intention of behavior.

This study is an explorative investigation, aiming to reveal a relationship between personality traits and the intention to show empathic behavior. To answer this question, we performed this study which gauged the correlation between General Intention to exhibit empathy and student personality. Additionally, we evaluated the influence of other factors, such as age, gender, curricular progress (second versus fourth year), and preferred specialization after graduation.

Seitz et al. (2017) found a significant difference in the intention to exhibit empathy between second versus fourth-year students as well as a significant difference contingent on age of the student. This could be due to a number of factors: perhaps the students get more realistic as they progress through their curriculum, or the intention statement of the second-year students was more a reflection of desire (a desire to be empathic) than a reflection of actual abilities, or students get more realistic with age, or they realize that treating patients medically does not entail empathy in every single action or situation (in giving an injection or during surgery, for example)—this list of potential explanations is by no means exhaustive. While there have been many studies investigating the impact of personality traits on the intention to show empathy in clinical settings (e. g., Austin et al. 2007), this current study is unique in that it concentrates on the preclinical terms, and it is expected that specific personality traits have an influence and further exploration is merited.

## Material and methods

### Participants

Medical students (*N* = 200) of the second and fourth year of the medical university of Vienna were asked to fill out two questionnaires (NEO-FFI and General Intention to Show Empathic Behavior (GISEB)) between two obligatory trainings on communication skills (Ärztliche Gesprächsführung). The participation in the study was voluntary. The study was accepted by the ethic committee of the Medical University of Vienna. The participants’ age range was 19 to 34 years; 90% were 26 years old or younger. Tables 1 and 2 give an overview on baseline data.

**Table 1:** Description of the sample

Participants N=131				
Academic year		Sex		NEO-FFI
2nd	4th	m	f	
N = 53	N = 78	N = 67	N = 64	N = 102
40.5%	59.5%	51.1%	48.9%	77.9%

**Table 2:** Sample description: Results of the questionnaires General Intention (GISEB) and NEO-FFI

	GISEB General Intention	NEO-FFI Neuroticism	NEO-FFI Extraversion	NEO-FFI Openness	NEO-FFI Agreeableness	NEO-FFI Conscientiousness
*N*	122	102	102	102	102	102
Mean (M)	5.86	1.76	2.47	2.65	2.71	2.7
Standard-deviation (SD)	1.11	.615	.61	.63	.51	.69
Variance	1.23	.38	.38	.40	.26	.47

### Measurements

#### NEO-FFI

The questionnaire (McCrae and Costa 2012) has shown good reliability and validity (Melchers et al. 2016). It consists of 60 questions, summarized in the following five categories:

1. Neuroticism: Measurement of emotional stability/lability. A person with high scores in this category is expected to be emotionally involved and overwhelmed easily in emotionally challenging situations, exhibiting less self-control than those with low scores.
2. Extraversion: Introverted persons described themselves as reserved, independent, and preferred to be alone, while extraverted people are rather active, talkative, social, and optimistic.
3. Openness: Persons with high scores describe themselves as curious, intellectual, imaginative, and adventurous, while persons with low scores prefer a rather conservative attitude.
4. Agreeableness: Persons with high scores aim for harmony, are flexible and trusting, while persons with low scores are competitive, suspicious and self-centered.
5. Conscientiousness: Measurement of performance-related self-control. Persons with high scores described themselves as ambitious, purposeful, reliable, and persistent, while persons with low scores did not have these character traits.

The reliability of the NEO-FFI is indicated by the internal consistencies of the five scales, which have a Cronbach alpha between *α* = .72 and *α* = .87. The Re-Test Reliabilities with a temporal difference of 5 years are between *r* = .71 and *r* = .82. With respect to the validity of the inventory, factor analyses alone and paired with other personality questionnaires show good construct validity (*r* = .54 to *r* = .80) (Borkenau and Ostendorf 2008). A translated version of the questionnaire was used (Körner et al. 2002).

### General Intention to Show Empathic Behavior

The questionnaire we used, adapted from “Constructing questionnaires based on the theory of planned behaviour” from Francis et al. (2004), consisted of three questions^1^ regarding the student’s intention and tendency to consistently demonstrate empathy. Intention was measured via questions with verbs such as “tend to,” “would like to,” and “plan to” show empathic behavior (German: tendiere, möchte versuchen, plane). According to Ajzen (1991), intention is to show how strongly a person is motivated to demonstrate a specific behavior and how much effort it takes to implement it (see Armitage and Conner 2001). In general, it can be assumed that the stronger the intention, the greater the likelihood of the intended behavior (see Ajzen 1991; Mattarelli 2007). The questions had to be answered on a seven-point Likert scale. Test criteria for this questionnaire as well as for the items relevant for measuring general intention were good: several categories reached a Chronbach *α* = .82 (.78 to .87); details on item specifity is given in Gruber (2015), corrected discriminatory power for the General Intention (GISEB) items *r*_it_ = .73 (.67 to .81). The detailed questionnaire and item formulations are presented in a pilot study (Seitz et al. 2017).

### We also asked for demographic data and wish of specialization.

### Statistical methods

We initially examined correlations between general intention to show empathy and gender, preferred specialization, and curricular progress (second versus fourth year). For all correlations, we performed sensitivity analyses using *t* tests or *u* test, depending on the distribution of the data. The evaluation of the normal distribution was performed using a Kolmogorow-Smirnow test. Next, we investigated the relationship between GISEB, personality (NEO-FFI), and age using Pearson’s or Spearman’s correlations. We used multivariate regression analysis with the variables gender, age, preferred specialization, and study progress and the NEO-FFI dimensions to predict general intention to show empathic behavior, respectively. The aim of the regression calculation was to identify the most important predictors for general intention and to account for multicollinearity between the five dimensions of the NEO-FFI. A 5% significance level was assumed for all tests. Analyses were performed using SPSS Statistics for Windows Version 22.0.

A post hoc power analysis for the correlation was conducted using the software G*Power (Faul et al. 2014). The sample size (*N* = 100) and the alpha level of *p* < .05 was used for the statistical power analyses (Faul et al. 2007). The recommended effect sizes used for this analysis were as follows: small (*r* = .10), medium (*r* = .30), and large (*r* = .50) (see Cohen 1992). The post hoc analyses revealed that the statistical power for this study was .17 for detecting a small effect, whereas the power exceeded .87 for the detection of a moderate effect and .99 for the detection of a large effect.

## Results

The response rate was 65.5%. One hundred thirty-one questionnaires were returned: 54 from the second-year students and 78 from the fourth year. Not all questionnaires were filled out completely. Therefore, *N* = 100 could be taken for calculation, and the mean age of the participants was 23–24 years (range 19–34), and the gender distribution was quite equal (male/female = 50.8:48.5%) (Table 1).

Of the students, 35.6% did not list which specialization they planned on pursuing after graduation. Of the rest, 29.4% reported a desire to specialize in Internal Medicine, 24.7% in Surgery, 12.9% in Pediatrics, 8.2% in Psychiatry, 4.7% in Neurology, 3.5% in General Medicine, 3.5% in Dentistry, 3.5% in Radiology, 2.4% in Anesthesia, 2.4% in Ophthalmology, 2.4% in Dermatology, one person in Rehabilitation, and another in General Research. Compared to the second year, significantly more students of the fourth year preferred to specialize in Internal Medicine (32 versus 25%) and fewer in Pediatrics (0.07 versus 21.8%). Gender-specific differences could be shown, too. Significantly more men wished to pursue surgery or psychiatry while significantly more women wished to pursue pediatrics.

### NEO-FFI and General Intention to Show Empathic Behavior

The exact results are listed in Table 2.

#### Influence of gender, age, curricular progress, and preferred specialization

##### Gender

No gender-specific differences could be shown regarding GISEB (*U* = 1496.5, *p* = .82) or NEO-FFI categories, except Neuroticism (*t* = − 4.662, *p* = .001), where women showed a significantly higher score.

##### Age

A significant but weak negative correlation between increasing age and GISEB was calculated (*r* = − .202, *p* = .026).

##### Study progress

A significant decrease of General Intention (GISEB) could be shown (*U* = 1203.5, *p* = .002) from second to fourth year of study.

##### Preferred specialization

The personality traits Conscientiousness (*F*(12, 53) = 2.015, *p* = .041) and Agreeableness (*F*(12, 53) = 2.376, *p* = .016) impacted the preferred specialization. The student who scored highest in Conscientiousness (*m* = 3.75) and Agreeableness (*m* = 3.08) expressed an interest in working in Rehabilitation. However, one respondent was statistically inconclusive and was thus excluded. The highest average score in Conscientiousness was achieved by students who wanted to specialized in General Medicine (*m* = 3.17) and Surgery (*m* = 2.91), the lowest in Radiology (*m* = 2.08) and Ophthalmology (*m* = 1.33). The highest average score in Agreeableness was achieved by students who wanted to specialized in General Medicine (*m* = 2.97) and Dentistry (*m* = 2.97), the lowest in Neurology (*m* = 2.29) and Surgery (*m* = 2.11).

#### Correlation between NEO-FFI and General Intention to Show Empathic Behavior

Significant but weak correlations were found between GISEB item tend to and Extraversion (*r* = .221, *p* = .027), as well as with Agreeableness (*r* = .229, *p* = .022), and between GISEB item plan to and Extraversion (*r* = .203, *p* = .043).

Recalling the post hoc power analyses which revealed low power for detecting small effects, it is assumed that these findings would have a stronger level of significance given more statistical power. Anyway, a correlation of about .2 is small (Cohen 1992) (Table 3).

**Table 3:** Correlations and 95% confidence intervals between General Intention to Show Empathic Behavior (GISEB) and NEO-FFI (*N* = 100)

	GISEB-1: “tend to”	GISEB-2: “like to try”	GISEB-3: “plan to”	NEO-FFI Neuroticism	NEO-FFI Extraversion	NEO-FFI Openness	NEO-FFI Agreeableness	NEO-FFI Conscientiousness
GISEB-1: “tend to”		.635** [.500–.750]	.554** [.388–.701]	− .055 [− .278–.163]	.221* [.013–.394]	.118 [− .068–.330]	.229* [.021–.428]	.058 [− .150–.265]
GISEB-2: “like to try”			.794** [.655–.900]	− .018 [− .211–.195]	.145 [− .053–.329]	− .030 [− .191–.169]	.123 [− .092–.332]	.049 [− .161–.263]
GISEB-3: “plan to”				− .062 [− .239–.140]	.203* [.008–.383]	− .042 [− .214–.165]	.117 [− .091–.324]	.076 [− .127–.279]
NEO-FFI Neuroticism					− .233* [− .439–− .021]	.098 [− .111–.286]	− .083 [− .289–.121]	− .236* [− .409–− .034]
NEO-FFI Extraversion						.181 [− .006–.370]	.258** [.047–.445]	.303** [.107–.487]
NEO-FFI Openness							.122 [− .070–.326]	− .118 [− .294–.060]
NEO-FFI Agreeableness								.212* [.013–.400]
NEO-FFI Conscientiousness								

*p < .05 (two-sided); **p < .001 (two-sided)

### Regression analysis

In the regression analysis, none of the included variables (age, sex, study progress, preferred specialization, and the NEO-FFI categories) were significant except study progress (*β* = − .27). This variable predicted weakly the general intention of planning to show empathic behavior (Table 4).

**Table 4:** Regression analysis

	*B*	*SE B*	*β*	Sig.
Constant	11.54	2.10		.000
Age	− .09	.07	− .17	.221
Study progress	− .41	.20	− .27	.049
Sex	.75	.46	.25	.098
Preferred Specialization	− .04	.04	− .13	.287
NEO-FF-I Neuroticism	− .66	.39	− .26	.09
NEO-FF-I Extraversion	.19	.37	.07	.62
NEO-FF-I Openess	− .36	.34	− .13	.29
NEO-FF-I Agreeableness	.28	.40	.10	.48
NEO-FF-I Conscientiousness	− .52	.31	− .22	.10

Note: *R*^2^ = .32

## Discussion

Our study showed only a few small correlations between the personality traits Neuroticism, Extraversion, Openness, Agreeableness, and Conscientiousness (NEO-FFI) and the student’s GISEB. Although extraverted persons seemed to have a greater intention to show empathic behavior, the personality trait itself was shown to be no predictor for General Intention (GISEB). Measured with these questionnaires, this means that the intention to show empathic behavior is not influenced by personality traits. Our results are not necessarily in conflict with the literature (Del Barrio et al. 2004; Nettle 2007; Magalhães et al. 2012; Lourinho and Severo 2013; Costa et al. 2014; Wakabayashi and Kawashima 2015; Melchers et al. 2016), which showed an association between personality and empathy. It might be that persons with specific personality traits vary in *showing* empathy even though the *intention* might be equal. In light of this distinction, these are encouraging results. With comprehensive and regular communication training of empathy, certain disadvantages due to personality may be ameliorated.

Another important finding is the decrease of general intention to show empathic behavior as students’ progress through their curriculum. These findings are in keeping with the literature which shows a decrease in empathy over the course of the curriculum, especially after the first practical experiences (Newton et al. 2008; Hojat et al. 2009; Colliver et al. 2010; Neumann et al. 2011).

Possible reasons might be the lack of models (Seitz et al. 2017) or the increasing stress (Neumann et al. 2011; Seitz and Löffler-Stastka 2016) that accumulates over the course of students’ studies. Other reasons might be students’ insecurity, feelings of being overwhelmed in working with clients, or the overload of processing client feelings (Pololi et al. 2001; Seitz and Löffler-Stastka 2016). This might lead to social withdrawal and the halting of prosocial behavior (Pololi et al. 2001; Lamm et al. 2007).

In our study, no gender-specific difference regarding General Intention to Show Empathic Behavior could be shown. However, literature shows differences in actual behavior. Female students and physicians on average have longer conversations with patients (Roter et al. 1997; Bylund et al. 2008; Löffler-Stastka et al. 2016) and include the psychosocial situation of the patient more often (Roter et al. 1997; Löffler-Stastka et al. 2016) compared to male colleagues. Additionally, women show a stronger non-verbal communication style (Roter et al. 1997; Bylund et al. 2008). However, it should be considered that the female students in our study showed higher scores in Neuroticism, suggesting the tendency to feel emotionally involved more easily. Some authors (Gleichgerrcht et al. 2013) support this thesis. As a result, women and men may have the same intention to show empathy, but due to gender-specific differences in the personality, women show more empathy towards patients.

The fourth interesting finding in our study is the influence of gender and personality in the students’ wish for specialization after graduation. This topic has already been discussed in several publications (McGrath and Zimet 1977; Wallick et al. 1999; Buddeberg-Fischer et al. 2003; Buddeberg-Fischer et al. 2006; Pawełczyk et al. 2007; Hojat and Zuckerman 2008; Malhi et al. 2011; Rotge et al. 2015). It is also interesting that the intended specialization typically changes after practical training in the fourth year. More students were interested in Internal Medicine than Pediatrics, showing that a lot of students may have had an inaccurate picture of their specific desired specialization in their first years of university. The influence of age regarding the general intention is most likely explained by the higher study year with increasing age.

## Conclusion

Our study revealed that although literature shows an association between empathy and personality, the intention to show empathic behavior is not influenced by personality traits measured by the NEO-FFI. This emphasizes the importance of comprehensive and regular communication and empathy training at Medical University Vienna. In the framework of this training, gender-specific and personality-related differences should be discussed. For future studies, it would be useful to evaluate how to successfully include such topics in the training.

## Footnotes

1 The questions were

1. I tend to show always empathic behavior in a situation of interaction with an (actor-)patient.
2. I would like to try to show always empathic behavior in a situation of interaction with an (actor-)patient.
3. I plan to show always empathic behavior in a situation of interaction with an (actor-)patient.

The original questions were in German:

1. Ich tendiere dazu, in einer Gesprächssituation mit (Schauspiel-) PatientInnen immer empathisches Verhalten zu zeigen (1 bis 7).
2. Ich möchte versuchen, in einer Gesprächssituation mit (Schauspiel-) PatientInnen immer empathisches Verhalten zu zeigen (1 bis 7).
3. Ich plane, in einer Gesprächssituation mit (Schauspiel-) PatientInnen immer empathisches Verhalten zu zeigen (1 bis 7).
